# Pneumorrhachis Secondary to a Locally Advanced Rectal Cancer with Pre-Sacral Abscess—Case Report and Review

**DOI:** 10.3390/medicina59122076

**Published:** 2023-11-25

**Authors:** Razvan Diaconescu, Dorel Popovici, Cristi Tarta, Alexandru Isaic, Dan Costachescu, Bogdan Totolici

**Affiliations:** 1 Department of Oncology, Faculty of Medicine, Victor Babeş University of Medicine and Pharmacy Timisoara, Eftimie Murgu Square 2, 300041 Timisoara, Romania; razvan.diaconescu@umft.ro (R.D.); dorel.popovici@umft.ro (D.P.); 2 Departments of General Surgery, Vasile Goldiş Western University of Arad, 310025 Arad, Romania; totolici_bogdan@yahoo.com; 3 Department X, 2nd Surgical Clinic, Researching Future Chirurgie 2, “Victor Babes” University of Medicine and Pharmacy Timisoara, Eftimie Murgu Sq. No. 2, 300041 Timisoara, Romania; isaic.alexandru@umft.ro; 4 Discipline of Radiology and Medical Imaging, “Victor Babes” University of Medicine and Pharmacy, 300041 Timisoara, Romania; costachescu.dan@umft.ro

**Keywords:** pneumorrhachis, intraspinal air, rectal fistula, rectal abscess, neuro-enteric fistula

## Abstract

The occurrence of pneumorrhachis (PR), defined as the presence of air within the spinal canal, presents a complex clinical picture with diverse etiological factors. We report an exceedingly rare case of PR arising from locally advanced rectal cancer accompanied by a pre-sacral abscess. This report aims to enhance awareness and understanding of rare causes of PR within the medical community, particularly among surgeons engaged in emergency procedures. The patient survived the acute phase of the disease through multiple surgical interventions and admission to the intensive care unit, but succumbed to cardiovascular complications three weeks later. We also offer a brief review of the literature concerning PR originating from the colorectal lumen.

## 1. Introduction

Pneumorrhachis (PR), defined as the presence of intraspinal gas, is a rare clinical and radiological manifestation with multifactorial etiologies, predominantly of traumatic, iatrogenic [1], and rarely infectious origin. Infectious PR could develop because of digestive–spinal fistula. This entity is observed concomitantly with a diverse array of pathological conditions and various mechanisms for gas introduction into the vertebral canal. This pathology was documented primarily within radiographic scholarly work; practitioners specializing in general surgery remain relatively unfamiliar with this atypical pathologic condition [2].

Building on a survey of existing medical literature in conjunction with an individual case study, we present a very rare case of PR due to a locally advanced rectal cancer with pre-sacral abscess and the decision-making algorithm, thereby enhancing awareness and understanding of very rare causes of pneumorrhachis within the medical community, especially among the surgeons involved in emergency surgery.

## 2. Case Report and Literature Review

### 2.1. Case Report

A 63-year-old female patient was admitted to the emergency department presenting with a constellation of symptoms indicative of severe systemic illness, including pyrexia with a temperature of 38.8 °C, chills, pallor, significant weight loss of fifteen kilograms over the preceding three months, and rectal hemorrhage. Additionally, she exhibited malodorous discharge from the perineal region and fecal incontinence. The acute symptom prompting her emergency visit was the sudden onset of bilateral lower extremity weakness, experienced earlier the same day while attempting ambulation; however, she arrived at the emergency department ambulatory. The patient’s medical history was notable for uncontrolled hypertension, for which she was non-compliant with prescribed antihypertensive therapy, and chronic rectal bleeding that had commenced several years prior. She was also identified as a heavy consumer of alcohol and tobacco.

The clinical examination revealed a lucid female patient presenting with pallor, tachycardia at a rate of 106 beats per minute, and a blood pressure reading of 110/60 mmHg; her breath exhibited an odor indicative of recent alcohol consumption, which she subsequently denied. She showed progressive difficulty in maintaining an upright posture, a clinical presentation that was attributed to a combination of anemia, dehydration, and sepsis. She also reported experiencing chronic perineal discomfort.

**Figure 1:**
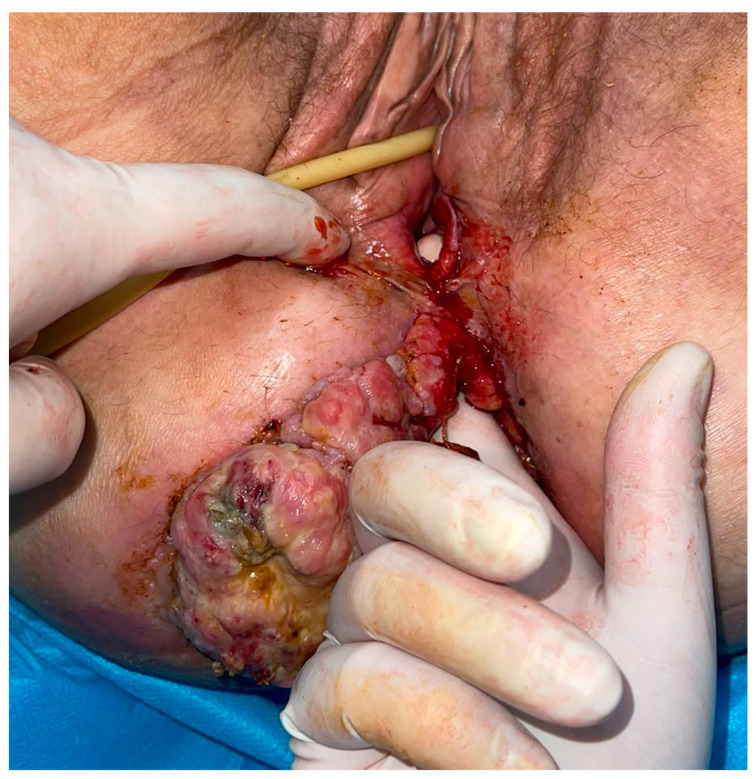
Rectovaginal fistula and perineal metastasis of rectal cancer.

On further inspection, the perineal area was observed to be compromised by an expansive neoplastic mass extending cranially towards both the rectal and vaginal canals, with evidence of a recto-vaginal fistula. The neoplasm was noted to be infected, malodorous, and hemorrhagic, with fecal matter identified within the vaginal tract (Figure 1).

Blood tests showed anemia (hemoglobin of 5 g/dL, hematocrit of 16.7%), leukocytosis of 21.130/mL with 92% neutrophils, albumin of 1.5 g/dL, and platelets at 524.000/mL.

**Figure 2:**
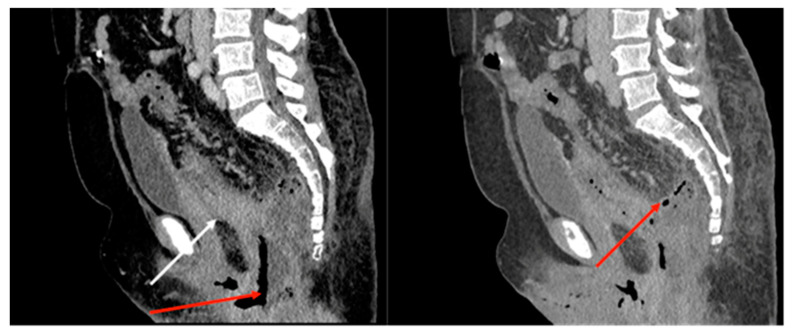
Sagittal CT of the pelvis; there is a rectal mass (white arrow) with an accompanying abscess (red arrow).

**Figure 3:**
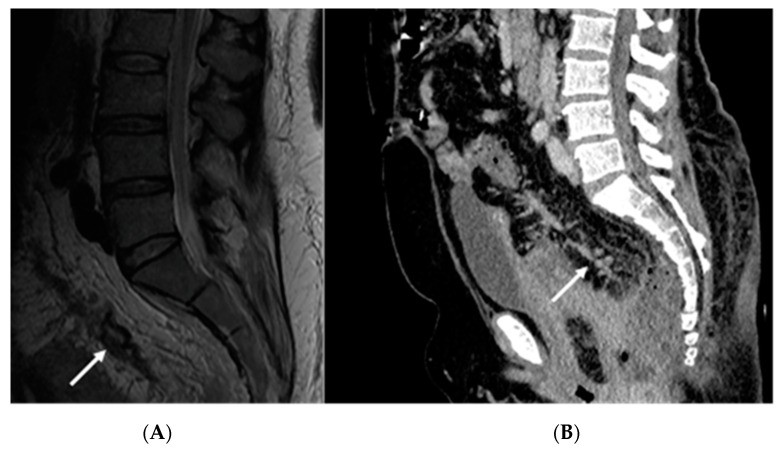
Sagittal T2 MRI (**A**) and sagittal CT image (**B**) depicting vascular invasion of the rectal tumor (white arrows).

**Figure 4:**
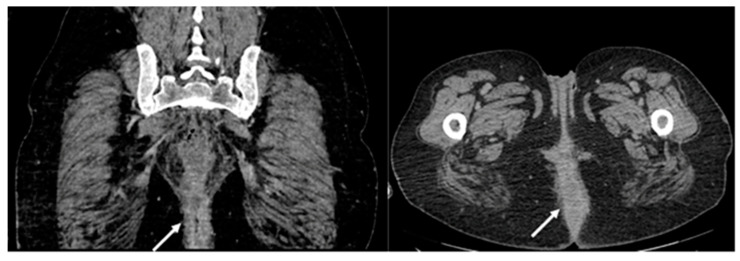
Pelvic CT depicting perineal invasion of the tumor (write arrows).

**Figure 5:**
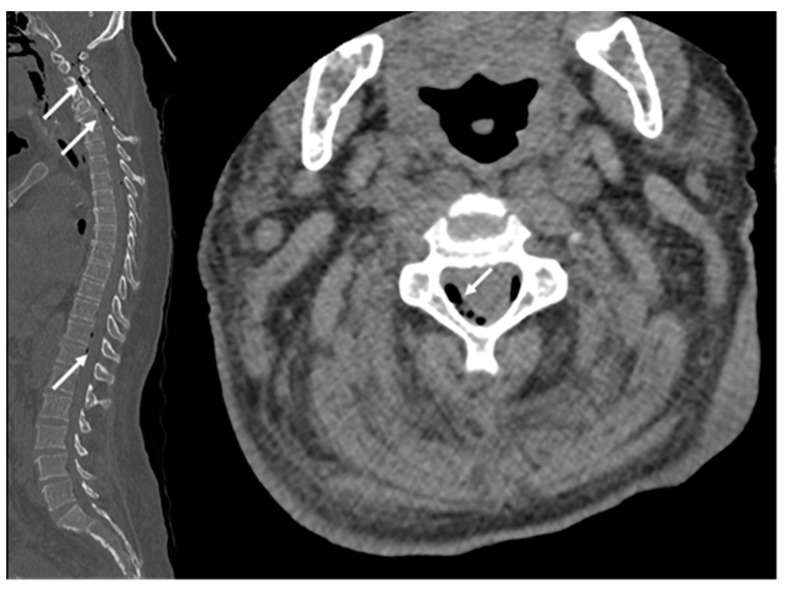
Pneumorrhachis (white arrows: extradural air bubbles are seen at the level of the cervical, thoracic, and lumbar spine.

A computed tomography (CT) scan was conducted, revealing a rectal neoplasm exhibiting local invasion into the uterine tissue and the posterior vaginal wall. Additionally, the imaging demonstrated a retrorectal abscess characterized by the presence of both air and fluid components, as well as pneumorrhachis (Figure 2, Figure 3, Figure 4 and Figure 5).

A diagnosis of perforated locally advanced rectal carcinoma, staged as cT4bN2M1, was established, accompanied by a retrorectal abscess and recto-vaginal fistula. Secondary diagnostic considerations included pneumorrhachis, sepsis, anemia, fecal incontinence, chronic alcohol abuse, heavy tobacco use, and uncontrolled hypertension. Neuro-logical manifestations at the time of presentation were minimal, characterized by postural instability and dizziness.

Given the patient’s critical condition and the extensive local tumor invasion, the im-mediate surgical strategy entailed abscess drainage, tumor biopsy, and the creation of a terminal colostomy to mitigate the risk of fecal contamination in the affected region (as the tumor was not causing an obstructive phenomenon). Intensive care interventions were initiated, comprising blood transfusion, fluid resuscitation, broad-spectrum antibiotic administration, and analgesic management. This acute management was intended to stabilize the patient in preparation for neo-adjuvant therapies, followed by definitive surgical intervention involving posterior pelvectomy and potential perineal reconstructive procedures.

During the initial resuscitation period spanning a few hours, the patient displayed isolated lower extremity weakness without additional neurological manifestations, rendering her unable to maintain an upright position.

Subsequently, the patient was transported to the operating theater where a laparoscopic end-colostomy was executed at the level of the proximal sigmoid colon. Intraperitoneal adhesions were noted within the pelvic cavity, though there were no discernible indicators of intra-abdominal infection. Drainage of the retrorectal abscess was facilitated via the rectal route, and a double-lumen Foley catheter was inserted to enable ongoing irrigation and drainage of the affected cavity. Multiple biopsy specimens were harvested for further pathological analysis and pus was sent to the lab in order to find the bacteria causing the abscess.

The postoperative trajectory was characterized by episodes of altered mental status and a progressive decline in lower extremity muscle strength, which was subsequently followed on the third postoperative day by compromised upper limb strength.

Neurological evaluation disclosed bilateral motor weakness affecting both the upper and lower extremities, accompanied by a generalized attenuation of sensory function in the lower extremities. Hyporeflexia was observed in deep tendon reflexes across both the upper and lower limbs, and ankle jerk reflexes were notably absent bilaterally. Given the presence of intraspinal air, as evidenced by pneumorrhachis, there was suspicion of a potential communication with the retrorectal abscess or the possibility of an anaerobic spinal infection with disseminated microorganisms originating from the abscess. 

From the lab we found that Escherichia coli was the bacteria from the abscess, and blood cultures were negative.

On the fifth postoperative day, the patient exhibited complete loss of limb function. Following a review of pertinent medical literature, the clinical decision was made to proceed with surgical intervention to eliminate the potential source of spinal contamination, even at the risk of achieving only a subtotal oncological resection of the rectal carcinoma.

Under general anesthesia, an abdominoperineal resection was performed through a midline xiphoid to pubic laparotomy, encompassing an en-bloc hysterectomy, bilateral adnexectomy, and posterior vulvectomy; an excision of involved cutaneous and subcutaneous perineal tissues was also done. Hemostasis was challenging to maintain owing to local inflammatory changes and neoplastic invasion, necessitating the packing of the pelvic and perineal regions with hemostatic sponges and patches and sterile gauzes. Subsequent to the operation, the patient was transferred to the intensive care unit due to breathing instability and was mechanically ventilated.

Within the first 24 h postoperatively, the patient required emergent reoperation owing to ongoing hemorrhage. Despite an exhaustive search, no identifiable source of bleeding was located. Hemostatic agents in the form of sponges and patches were deployed, and repacking of the pelvic and perineal regions was performed to control the hemorrhage.

Forty-eight hours post-procedure, the patient regained consciousness and displayed bowel activity via the colostomy. She was hemodynamically stable and was utilizing a facial oxygen mask for respiratory support. The upper limb motor function was partially restored, enabling her to manipulate objects with her hands. Sensation was present in the lower extremities, although motor function remained compromised. The abdominal packing was extracted on the third postoperative day, with concurrent replacement of perineal packing. This perineal packing was subsequently removed on the sixth postoperative day and replaced with sterile gauze.

Systemic antibiotic therapy was perpetuated, paralleled by incremental recuperation of lower extremity motor function. By the seventh postoperative day, the patient regained the ability to move her feet. Subsequent neurosurgical and neurological assessments delineated the ongoing therapeutic needs and tracked the patient’s progressive neurological recovery.

Regrettably, the patient succumbed suddenly on the 25th day post-admission, the cause of which was presumptively ascribed to cardiorespiratory arrest, likely secondary to a pulmonary embolism. A post-mortem examination was not conducted, as local legislation permitted patient relatives to decide to forgo this procedure. Prior to her demise, the patient had mostly recovered upper extremity motor function and displayed the ability for foot movement and sustained knee flexion, but was not able to stand or walk. 

**Figure 6:**
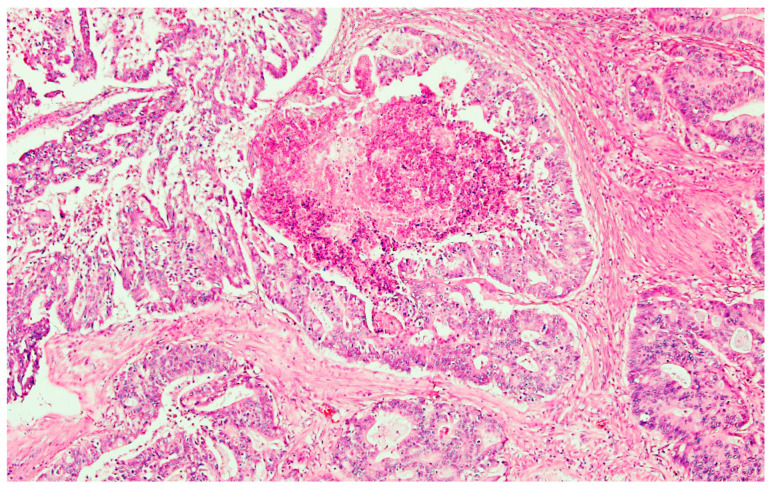
Adenocarcinoma NOS with dirty necrosis. He × 200.

**Figure 7:**
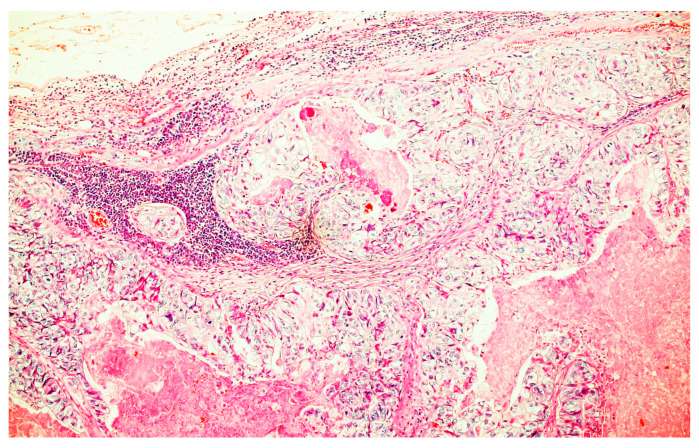
Lymph node metastasis of the tumor. He × 200.

The pathology report showed rectal cancer stage IV A with T4bN2bLV2Pn1M1aR1 (Figure 6 and Figure 7).

### 2.2. Review of the Literature

Inclusion criteria were as follows: papers reporting cases with pneumorrhachis with the origin at the level of a colorectal abscess, written in the English language, and with full text availability. A systematic review was conducted on the PubMed database (https://pubmed.ncbi.nlm.nih.gov/) (searched on 5 February 2023) utilizing a search syntax composed of the following terms: ((((((((((((((intraspinal pneumocele) OR (pneumocoele)) OR (spinal epidural pneumatosis)) OR (subarachnoid pneumatosis)) OR (spinal emphysema)) OR (epidural emphysema)) OR (aerorachia)) OR (pneumosaccus)) OR (air myelogram)) OR (pneumomyelogram)) OR (pneumomyelography)) OR (pneumocephalus)) OR (pneumorrhachis)) OR (pneumorrachia)) AND (abscess). The synonym terms for pneumorrhachis were the ones found in the review by Ortel et al. [1]. The literature search yielded 112 articles. Two investigators (RD and CT) meticulously screened these articles by scrutinizing either the abstracts or the full-length manuscripts when abstracts were unavailable, to ascertain their relevance for inclusion in this review. Subsequent reference tracing was conducted to identify additional pertinent publications. One additional article was discovered through this reference search. Ultimately, six articles met the criteria for inclusion in this review, and one was excluded due to unavailability of the full text [3,4,5,6,7]. Notably, all five articles were case reports discussing pneumorrhachis as a sequela of colonic conditions, as summarized in Table 1.

The included case reports featured three instances of perforated diverticulitis, one case of postoperative fistula formation, and one case following rectal surgery involving a chronically infected presacral sinus, which manifested eight months following loop ileostomy closure. Surgical interventions aimed at sepsis control uniformly involved segmental resections followed by end colostomies. All patients manifested pneumocephalus and pneumorrhachis, accompanied by neurological deficits, pyrexia, chills, and clinical indications of meningitis. Of the five cases, four patients survived: one exhibited mild residual left-sided hemiparesis at a two-year follow-up evaluation. The outcome for one patient was not specified in the corresponding publication.

## 3. Discussion

The occurrence of pneumocephalus and pneumorrhachis (PR), defined, respectively, as the presence of air within the cranial vault and the spinal canal, offers a complex clinical tableau with diverse etiological underpinnings [1]. The present cases exhibit the confluence of these phenomena with other severe medical conditions, such as locally advanced rectal carcinoma, diverticulitis, and pre-sacral abscesses, further complicating diagnostic and therapeutic decision-making.

Two principal pathways may facilitate the entry of air into these neural structures: a breach in the dural nerve sheath of the sacral nerve plexus and the pre-sacral venous plexuses [8,9]. The former has been described as an uncommon complication in the context of metastatic rectal adenocarcinomas with adjacent osseous erosion [9]. This breach allows air to seep into the subarachnoid spaces of both the cranial and spinal cavities.

The diagnostic landscape for pneumorrhachis (PR) is continually evolving, with advanced imaging modalities offering unprecedented insights into this rare but clinically significant phenomenon. Computed tomography (CT) remains the gold standard for diagnosing PR, given its high sensitivity and specificity in detecting even trace amounts of air within the spinal canal. Moreover, CT scans provide valuable anatomical details that can be critical for surgical planning and for discerning potential etiologies, such as breaches in the dura or vascular anomalies [10].

However, the utility of magnetic resonance imaging (MRI) should not be discounted. While generally less sensitive than CT for detecting air, MRI offers superior soft tissue contrast and can be invaluable for identifying concurrent abnormalities, such as spinal cord compression or intramedullary pathology. Additionally, MRI poses fewer risks related to radiation exposure, a non-trivial consideration in certain populations, such as pregnant women and pediatric patients [11]. MRI can predict local difficulties and anatomical challenges in patients with colorectal cancer both for open or minimally invasive surgery by approximating the size of the mesorectum and bony structures [12]. There are papers which tend to favor the robotic approach for rectal cancer, especially for difficult cases with a narrow pelvis, high body mass index, and/or male sex, but this requires proficiency in minimally invasive surgery and an experienced surgeon, and in our case, we could not risk introducing more air in the spine by insufflating pneumoperitoneum [13].

Despite these advanced imaging techniques, diagnostic challenges persist, particularly in the context of co-existing pathologies such as tumors or infections, which can obscure the presentation. A multidisciplinary approach, incorporating both radiological and clinical assessments, is therefore paramount for accurate diagnosis and subsequent management [1,4].

In summary, while CT remains the primary imaging modality for diagnosing PR, the role of MRI is increasingly recognized, and a nuanced, individualized diagnostic strategy is essential for optimal patient care as seen in our review; more recent studies used both methods [6,7].

The Hartmann procedure was employed in all the cases. This procedure has several advantages—while removing the affected part of the bowel, it lowers the possibility of an anastomotic fistula which poses a threat to the patient’s life after surgery, a very important aspect for those patients with severe neurological impairment. None of the identified studies mentioned a reversal of the procedure. Recent studies highlight a variable rate of Hartmann procedure reversals, ranging between 17% (colostomy for bowel ischemia) and 85% (colostomy after trauma) [14]. These percentages underscore the multifaceted nature of decision-making in colorectal surgery. Factors influencing these rates include patient comorbidities, age, initial disease severity, and postoperative complications. The literature also points to advancements in surgical techniques and postoperative care, potentially influencing these rates. However, the decision for reversal is complex, necessitating a balanced consideration of risks and benefits. This variability in reversal rates emphasizes the need for individualized patient evaluation and a nuanced approach to post-colorectal surgery care [14].

One of the reasons for our case outcome might be the amplitude of the surgery, the postoperative complications, and the presence of advanced cancer, as all the other cases involved benign disease or a long period after primary cancer surgery.

Given these multifaceted etiologies and routes of air entry, the cases underscore the essentiality of a nuanced, multidisciplinary approach involving gastroenterology, neurosurgery, oncology, and infectious diseases for optimal management. Monitoring subtle neurological symptoms remains crucial as these can serve as early indicators for immediate intervention, potentially averting further neurological compromise.

This clinical complexity is further amplified when PR and pneumocephalus occur in the setting of severe systemic conditions such as malignancy and sepsis. Our findings, therefore, not only contribute to the growing body of literature, but also raise awareness for the risk factors for these conditions, particularly when complicated by malignancy and infection.

## 4. Conclusions

Our case exhibited several unique characteristics that complicated the formulation of a straightforward treatment trajectory, including locally advanced rectal carcinoma, the patient’s overall poor clinical condition, and the insidious onset of neurological symptoms. While we succeeded in mitigating the acute phase of the disease, the patient ultimately succumbed to systemic complications.

## Figures and Tables

**Table 1:** Case reports detailing pneumorrhachis as a consequence of colonic-associated abscesses.

Authors/Year	Sex/Age	Abscess Location	Pneumorrhachis	Imaging	Operation	Outcome
Krishna 1998 [3]	f/71	Sigmoid colon diverticulitis/lumbar 5th spine	Pneumocephalus and pneumorrhachis	CT	Hartmann procedure	Alive, recovered
Shetty 2007 [4]	m/48	Sigmoid colon diverticulitis/sacrum	Pneumocephalus and pneumorrhachis	CT	Hartmann procedure and ileostomy	Alive, recovered
Annaye 2008 [5]	m/80	Left colon anastomotic fistula with abdominal wall abscess	Pneumorrhachis and pneumoscrotum, pneumomediastinum, pneumothorax	CT	Hartmann procedure	Not stated
Warsi 2010 [6]	m/60	Chronic anastomotic rectal leak with pre-sacral abscess	Pneumocephalus and pneumorrhachis	CT, MRI	Hartmann procedure	Alive, recovered
Amit 2011 [7]	m/60	Sigmoid colon diverticulitis/sacral spine	Pneumocephalus and pneumorrhachis	CT, MRI	Hartmann procedure	Alive, mild residual left-sided hemiparesis

## Data Availability

Data are available upon request to the corresponding author.
